# Neoadjuvant chemoradiotherapy with or without PD-1/PD-L1 inhibitors in locally advanced rectal cancer: a systematic review and meta-analysis

**DOI:** 10.1186/s12885-025-14482-5

**Published:** 2025-07-01

**Authors:** Muhammad Ansab, Sepideh Razi, Farwa Nisa, Eiman Araib, Shree Rath, Iqra Khan, Noor Ul Huda Ramzan, Esha Dilawar, Anwaar Saeed

**Affiliations:** 1 Faculty of Medicine, Services Institute of Medical Sciences, Lahore, Pakistan; 2 Research Center for Immunodeficiencies, Children’s Medical Center, Tehran University of Medical Sciences, Tehran, Iran; 3 Faculty of Medicine, Fatima Jinnah Medical University, Lahore, Pakistan; 4 Faculty of Medicine, Dow University of Health Sciences, Karachi, Pakistan; 5 Faculty of Medicine, All India Institute of Medical Sciences, Bhubaneswar, India; 6 Faculty of Medicine, University Medical and Dental College, Lahore, Pakistan; 7 Department of Medicine, Division of Hematology & Oncology, University of Pittsburgh Medical Center (UPMC), 5150 Centre Ave, Pittsburgh, PA 15213 USA; 8 UPMC Hillman Cancer Center, Pittsburgh, PA 15213 USA

**Keywords:** Locally advanced rectal cancer, PD-1 inhibitors, Neoadjuvant chemoradiotherapy, Pathological complete response, Immunotherapy, Clinical complete response, Short-course radiotherapy

## Abstract

**Background:**

Locally advanced rectal cancer (LARC) represents a pivotal stage of rectal cancer where it is possible to completely cure the cancer before its systemic spread, thus often requiring an aggressive multimodal therapy. Recent trials suggest that programmed cell death protein 1 (PD-1)/programmed cell death ligand 1 (PD-L1) inhibitors combined with neoadjuvant chemoradiotherapy (CRT) may improve treatment outcomes. This systematic review and meta-analysis aimed to evaluate the efficacy and safety of PD-1/PD-L1 inhibitors when integrated into neoadjuvant CRT regimens for LARC patients.

**Methods:**

A systematic search of PubMed, Embase, ClinicalTrials.gov, and Cochrane Central Library was conducted up to October 11, 2024. Randomized controlled trials (RCTs) comparing neoadjuvant CRT with or without PD-1/PD-L1 inhibitors were included. The primary outcomes assessed were pathological complete response (pCR), clinical complete response (cCR), and serious adverse events (SAEs). Pooled odds ratios (ORs) with 95% confidence intervals (CIs) were calculated using a random-effects model. Subgroup analyses were performed to explore variations in radiotherapy strategies, types of PD-1/PD-L1 inhibitors used, and mismatch repair (MMR) status.

**Results:**

Six RCTs (861 patients) met the inclusion criteria. PD-1 inhibitors significantly improved pCR rates (OR = 2.10, 95% CI: 1.32–3.32, *p* = 0.001), particularly with short-course radiotherapy (SCRT) and agents like Camrelizumab and Tislelizumab. However, PD-1 inhibitors did not significantly enhance cCR (OR = 1.54, 95% CI: 0.51–4.63, *p* = 0.44) or increase SAEs (OR = 1.08, 95% CI: 0.73–1.60). Subgroup analysis based on MMR status revealed a significantly higher pCR rate in the proficient MMR (pMMR) subgroup compared to the deficient MMR (dMMR) subgroup, although the result for dMMR was non-significant due to limited sample size and the absence of reported events.

**Conclusions:**

The addition of PD-1 inhibitors to neoadjuvant CRT significantly improves pCR rates in LARC without increasing toxicity. These findings support their potential role in standard treatment protocols, warranting further phase III trials.

**Registration number:**

CRD42024619949.

**Supplementary Information:**

The online version contains supplementary material available at 10.1186/s12885-025-14482-5.

## Introduction

Colorectal cancer is one of the most commonly occurring malignancies, ranking third in prevalence and second in the number of deaths in the United States [1, 2]. Nearly 2 million cases were newly diagnosed in 2022. A growing prevalence among the younger population aged less than 50 years has been noted, increasing at a rate of 0.5–3% per year [3]. Despite the introduction of guidelines for occult blood examination and colonoscopy, a lack of global availability of screening resources and education remains a concern.

Colorectal cancer staging follows the TNM classification, with stages ranging from 0 to 4. Among these stages, locally advanced rectal cancer (LARC) refers to cases where a T3 or T4 tumor is observed or where there is node-positive disease but no metastasis [4]. LARC forms the critical point between local disease and metastasis and is the last stage that can potentially be completely cured with good survival outcomes.

Current guidelines for the management of LARC include a detailed pelvic Magnetic Resonance Imaging (MRI) scan to assess and confirm the stage of the tumor, as well as a genotypic assessment to evaluate mismatch repair (MMR) defects of microsatellite instability. Treatment options are guided mainly by the chromosomal defect. Those with positive MMR status are primarily treated with total neoadjuvant therapy (TNT), consisting of chemoradiotherapy (CRT). The standard “FOLFOX” regimen, along with radiation, is an example of neoadjuvant therapy. In those with high microsatellite instability, immunotherapy is the standard treatment adopted unless contraindicated [5]. With the current treatment guidelines, survival rates of 93% at 3 years and 81.6% at 5 years have been noted [6]. However, rates of distant metastases have remained constant over the years [7], suggesting that current therapies are still inadequate in completely eliminating cancer cells. Up to 1 in 3 patients with LARC may develop metastases, which significantly reduce overall survival (OS) and disease-free survival (DFS).

With the overall growing incidence of LARC, multiple therapies and drugs are being repurposed for use in specific malignancies. Another goal is to treat the vast majority of the population, including those with atypical presentations and genetic profiles. Among these drugs, the use of anti-programmed cell death protein 1 (PD-1) drugs has been evaluated for use in LARC. In a recent study by Coussement et al., high programmed cell death ligand 1 (PD-L1) expression was noted among rectal cancer cells and was associated with better outcomes post-operatively [8]. Better short-term outcomes have been noted among patients being administered anti-PD-1/PD-L1 antibodies [9, 10].

Following multiple hypotheses and cell-based findings, emerging trials have highlighted the efficacy of anti-PD-1/PD-L1 drugs in TNT. A Phase 2 study by Cercek et al. found a complete response (CR) among the 12 included patients who received the combination therapy [11]. Yang et al. noted similar findings among their cohort of patients, with lower rates of serious adverse events (SAEs) [12]. We aim to collate these findings in this systematic review and meta-analysis, which will quantify the efficacy of adding PD-1/PD-L1 inhibitors to neoadjuvant CRT regimens in patients with LARC. This study will help highlight the various benefits and pitfalls of this therapy and guide clinicians when formulating a treatment plan.

## Methods

This systematic review and meta-analysis follows the Preferred Reporting Items for Systematic Reviews and Meta-Analyses (PRISMA) guidelines (Supplementary Table 1) [13]. The protocol was registered in the International Prospective Register of Systematic Reviews (PROSPERO) under the registration number CRD42024619949.

### Data sources and search strategy

An electronic search of PubMed, Embase, ClinicalTrials.gov, and Cochrane Central Library was conducted from their inception to October 11, 2024, without any language restrictions, with additional relevant studies included up to the finalization of this manuscript. The search utilized the following keywords, such as “Rectal Neoplasms,” “Locally Advanced Rectal Cancer,” “LARC,” “Neoadjuvant Chemotherapy,” “Neoadjuvant Chemoradiotherapy,” “Nivolumab,” “Opdivo,” “PD-1 Inhibitors,” “Programmed Cell Death Protein 1 Inhibitors,” “Dostarlimab,” “Jemperli,” and “Sintilimab,” and combined them using AND or OR functions. The detailed search string for each database is provided in Supplementary Table 2. The reference lists of retrieved trials, previous meta-analyses, and review articles were manually screened to identify any relevant studies.

### Study selection

Only randomized controlled trials (RCTs) were selected for this review. Narrative and systematic reviews, posters, conference abstracts, case reports, letters to the editor, and other articles that did not meet the inclusion criteria were cross-referenced for additional potential sources of RCTs. Articles retrieved from the literature search were exported to Rayyan AI, where duplicates were identified and removed. Two reviewers (I.K. and N.R.) independently assessed the remaining articles, and only those trials that met the predefined criteria were selected. All trials were initially shortlisted based on title and abstract, followed by a full-text review to confirm relevance. A third investigator (F.N.) was consulted to resolve any discrepancies. The following inclusion and exclusion criteria were applied to select studies: RCTs involving patients with a confirmed diagnosis of LARC. These studies compared the outcomes of neoadjuvant CRT combined with PD-1/PD-L1 inhibitors versus neoadjuvant CRT alone. Only peer-reviewed articles and American Society of Clinical Oncology (ASCO) abstracts or conference proceedings with sufficient data for analysis were included. Studies were excluded if they were non-randomized or observational, involved other cancer types or stages, lacked PD-1/PD-L1 inhibitors in the intervention, were unpublished, or had incomplete outcome data. At the end of the selection process, six studies that met these criteria were included in the analysis.

### Data extraction

In this review, data extraction was systematically conducted to ensure accuracy and relevance. Key information was collected from each study, including study design, country of origin, intervention (e.g., type, dosage, and administration details), and comparator (e.g., placebo or standard treatments). Sample characteristics were also recorded, such as total population (intervention/comparator), gender distribution (number and percentage of males), and median age. Additionally, tumor details, including T category, N category, tumor location (proximal/distal), outcome measures (SAEs, pathological complete response (pCR), and clinical complete response (cCR)) and study results, were also extracted. MMR status, classified as proficient (pMMR) or deficient (dMMR), along with the corresponding number and percentage of participants, was reported. Two independent reviewers (E.D. and I.K.) performed data extraction to minimize bias, with discrepancies resolved through discussion or consultation with a third reviewer (M.A.). In one study [14], the number of patients who experienced SAEs in the experimental and control groups was unclear. The corresponding author was contacted to obtain this information, and the provided data were included in the analysis. Data were organized and recorded in a standardized Excel sheet to facilitate consistent comparison and synthesis across studies.

### Quality assessment and risk of bias

Cochrane Collaboration’s Risk of Bias 2 (ROB 2) [15] tool for RCTs was applied to assess the quality of the included trials. This tool evaluates domains such as D1 (bias arising from the randomization process), D2 (deviations from intended interventions), D3 (missing outcome data), D4 (measurement of the outcome), and D5 (selection of the reported result). Disagreements were discussed with the third investigator after two reviewers independently assessed the quality of the studies. Each of the six included studies was rated for potential bias, and the results were thoroughly reviewed to ensure all studies were appropriately considered in the overall analysis.

Due to the limited number of included studies, Funnel plots were not generated for the assessment of publication bias, and Begg’s test was not conducted as its results could be misleading. Instead, DOI plots were created, and the LFK indexes were calculated. The DOI identifies studies with a disproportionate influence on results; values outside the recommended range may require further investigation [16].

### Statistical analysis

All statistical analyses were conducted using R Studio (version 4.1.1). Results from trials were presented as odds ratios (ORs) with 95% confidence intervals (CIs) and pooled using a random-effects model. Forest plots were generated to visually represent the pooled results. Sensitivity analysis, including influence analysis by omitting one study at a time to assess the robustness of the synthesized results, was performed on the studies. Heterogeneity among studies was assessed using the I² statistic, which accounts for variability across studies. An I² estimate of 50% or higher suggests potential heterogeneity, while values between 75% and 100% indicate considerable heterogeneity. The I² statistic was calculated based on the χ² statistic (Q) and its degrees of freedom. Heterogeneity was further explored using methods developed by Baujat et al. [17] and Galbraith [18]. Galbraith plots were generated, where studies closer to the regression line indicated lower heterogeneity, while those farther away suggested greater variability. To assess the potential underlying causes of heterogeneity, exploratory subgroup analyses were done based on the radiotherapy strategy, the type of PD-1/PD-L1 inhibitor used, and MMR status. Additionally, subgroup analyses of SAEs based on the radiotherapy strategy and the PD-1/PD-L1 inhibitor used were conducted. A p-value of less than 0.05 was considered significant for all statistical tests.

## Results

### Search results

**Fig. 1:**
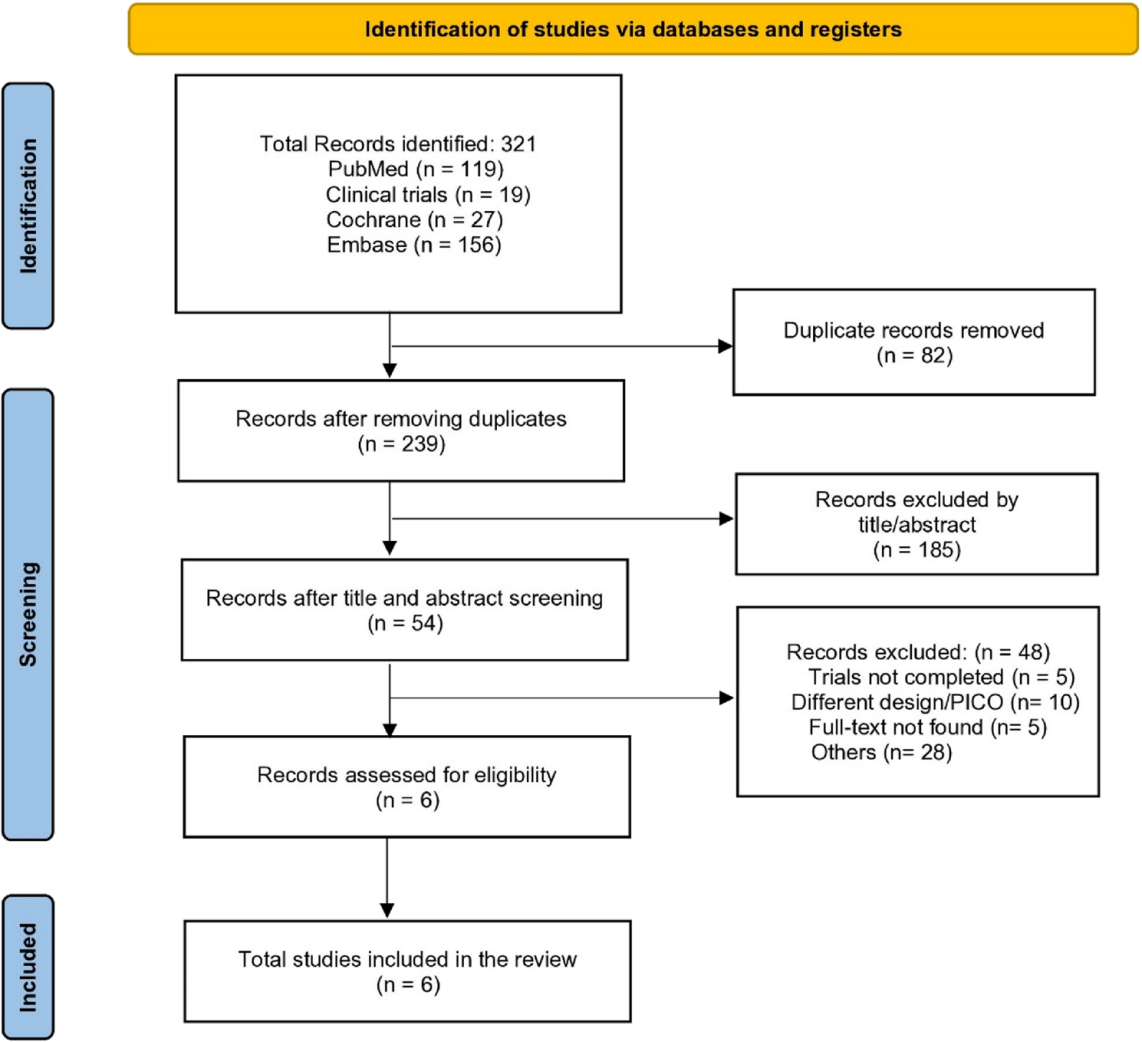
Preferred Reporting Items for Systematic Review and Meta-Analysis (PRISMA) flow diagram of study screening and selection

The initial search from databases, including PubMed, Embase, ClinicalTrials.gov, and Cochrane Central Library, yielded 321 records. After the removal of duplicates, 239 records were assessed based on their titles and abstracts. After the primary screening, 54 records were evaluated based on their full texts, and finally, 6 studies [14, 19–23] were considered eligible for inclusion in our systematic review and meta-analysis based on our predefined inclusion criteria. Details of article screening are given in the PRISMA diagram (Fig. 1).

### Patient characteristics and treatment details

Of the 6 included studies, all were RCTs. All except one [23] were based in China; the remaining study was based in the United States. There was a total of 861 patients with LARC in our study: 402 in the group receiving neoadjuvant CRT without a PD-1 inhibitor and 459 in the group receiving neoadjuvant CRT with a PD-1 inhibitor. PD-1 inhibitors included Sintilimab in two studies [14, 21], Tislelizumab in two studies [20, 22], and Camrelizumab and Pembrolizumab in the remaining two studies [19, 23]. The phase number of the RCTs, dosage and administration details of PD-1 inhibitors, chemotherapy strategy, radiotherapy details, and cancer characteristics such as T category, N category, tumor location (proximal or distal), and MMR status are provided in Table 1.

**Table 1:** Design and characteristics of studies included in the meta-analysis

Study	Year	Study Type	Phase	Country	Intervention	Comparator	Dosage details		Total population (I/C)	Male *n* (%) (I/C)	Median age (range) (I/C)	T Category *n* (%)		*N* Category *n* (%)		Tumor location Proximal/Distal		MMR Status *n* (%) Proficient/Deficient	
							Intervention	Comparator				Intervention	Comparator	Intervention	Comparator	Intervention	Comparator	Intervention	Comparator
Xiao et al. [14]	2024	RCT	II	China	Neoadjuvant Oxaliplatin and Capecitabine + LCRT + Sintilimab	Neoadjuvant Oxaliplatin and Capecitabine + LCRT	1 cycle of Oxaliplatin (130 mg/m² IV, day 1) and Capecitabine (1000 mg/m² BID, orally, days 1–14, q21d); then LCRT (45–50 Gy/25 fractions) + 2 cycles of Oxaliplatin (100 mg/m² IV, day 1) and Capecitabine (1000 mg/m² BID, orally, days 1–14, q21d); then 1 cycle of Oxaliplatin and Capecitabine (same dose as the first cycle); Sintilimab (200 mg IV, day 1, q21d) given with all 4 cycles; ± Surgery	1 cycle of Oxaliplatin (130 mg/m², IV, day 1) and Capecitabine (1000 mg/m² BID, orally, days 1–14, q21d); then: LCRT (45–50 Gy in 25 fractions); 2 cycles of Oxaliplatin (100 mg/m², IV, day 1) and Capecitabine (1000 mg/m² BID, orally, days 1–14, q21d); then: 1 cycle of Oxaliplatin and Capecitabine (same dose as first cycle); ± Surgery	134 (67/67)	43 (64.2)/37 (55.2)	56 (33–73)/56 (25–72)	T2 = 2 (3) T3 = 30 (44.8) T4 = 35 (52.2)	T2 = 2 (3) T3 = 33 (49.3) T4 = 32 (47.8)	N0 = 10 (14.9) N1 = 34 (50.7) N2 = 23 (34.3)	N0 = 8 (11.9) N1 = 27 (40.3) N2 = 32 (47.8)	23 (34.3)/44 (65.7)	25 (37.3)/42 (62.7)	67 (100)/0	67 (100)/0
Lin et al. [19]	2024	RCT	III	China	Neoadjuvant SCRT followed by Oxaliplatin and Capecitabine + Camrelizumab	Neoadjuvant LCRT followed by Oxaliplatin and Capecitabine	SCRT (25 Gy in 5 days), then Oxaliplatin (130 mg/m² IV over 2 h, day 1) and Capecitabine (1000 mg/m² BID, orally, days 1–14, q21d × 2) + Camrelizumab (200 mg IV, day 1; q21d × 2), starting 1 week later; Surgery within 10 weeks after radiotherapy; 4–6 weeks post-op: 6 cycles of Camrelizumab + Oxaliplatin and Capecitabine, followed by Camrelizumab alone	LCRT (50.4 Gy in 28 days) + Capecitabine (825 mg/m² BID, orally); 2 weeks later: 2 cycles of Oxaliplatin and Capecitabine; Surgery within 10 weeks after radiotherapy; 4–6 weeks post-op: 6 cycles of Oxaliplatin and Capecitabine	231 (113/118)	75 (66.4)/78 (66.1)	58 (32–75)/58 (31–75)	T2 = 7 (6.2) T3 = 72 (63.7) T4 = 34 (30.1)	T2 = 4 (3.4) T3 = 79 (66.9) T4 = 35 (29.7)	N0 = 14 (12.4) N1 = 48 (42.5) N2 = 51 (45.1)	N0 = 17 (14.4) N1 = 61 (51.7) N2 = 40 (33.9)	57 (50.4)/56 (49.6)	62 (52.5)/56 (47.5)	105 (92.9)/3 (2.7)	112 (94.9)/2 (1.7)
Li, W et al. [20]	2024	RCT	I/II	China	Neoadjuvant SCRT + Capecitabine and Oxaliplatin + Tiselezumab	Neoadjuvant SCRT + Capecitabine and Oxaliplatin	SCRT (25 Gy/5 fractions) + Capecitabine (1000 mg/m² BID, orally, days 1–14, q21d × 4) and Oxaliplatin (130 mg/m² IV, day 1) + Tislelizumab (200 mg IV, day 1)	SCRT (25 Gy/5 fractions) + Capecitabine (1000 mg/m² BID, orally, days 1–14, q21d ×4) and Oxaliplatin (130 mg/m² IV, day 1)	40 (21/19)	16 (76.1)/10(52.6)	NR	NR	NR	NR	NR	8 (38.1)/13 (61.9)	8 (42.1)/11 (57.9)	21 (100)/0	19 (100)/0
Li, H et al. [21]	2024	RCT	II/III	China	Neoadjuvant SCRT + Capecitabine and Oxaliplatin or Folinic Acid, Fluorouracil (5-FU) and Oxaliplatin + Sintilimab	Neoadjuvant SCRT + Capecitabine and Oxaliplatin or Folinic Acid, Fluorouracil (5-FU) and Oxaliplatin	SCRT (25 Gy in 5 fractions), then 4 cycles of Capecitabine and Oxaliplatin or 6 cycles of Folinic Acid, Fluorouracil (5-FU) and Oxaliplatin + Sintilimab; Surgery; 2 postoperative cycles of same regimen	SCRT (25 Gy in 5 fractions), then 4 cycles of Capecitabine and Oxaliplatin or 6 cycles of Folinic Acid, Fluorouracil (5-FU) and Oxaliplatin; Surgery; 2 postoperative cycles of same regimen	100 (54/46)	NR	NR	NR	NR	NR	NR	NR	NR	54 (100)/0	46 (100)/0
Yang et al. [22]	2025	RCT	II	China	Neoadjuvant CRT (Capecitabine) + concurrent or sequential Tislelizumab	Neoadjuvant CRT (Capecitabine)	LCRT (~ 45–50.4 Gy/~25–28 fractions) + Capecitabine (825 mg/m² BID, orally, 5 days/week for 5 weeks); then: Tislelizumab (200 mg, IV ×3 doses, every 3 weeks) (first dose given 1 week after radiotherapy start (concurrent) or 2 weeks after completion (sequential); Surgery 8–12 weeks after completion of radiotherapy	LCRT (~ 45–50.4 Gy/~25–28 fractions) + Capecitabine (825 mg/m² BID, orally, 5 days/week for 5 weeks); Surgery 6–12 weeks after completion of radiotherapy	171 (114/57)	77 (67.5)/41 (72)	NR	T1 = 1 (0.87) T2 = 7 (6.14) T3 = 77 (67.5) T4a = 29 (25.4)	T1 = 0 (0) T2 = 2 (4) T3 = 43 (75) T4a = 12 (21)	N0 = 14 (12.28) N1 = 47 (41.2) N2 = 53 (46.4)	N0 = 12 (21( N1 = 21 (37) N2 = 24 (42)	81 (71.05)/32(28.07)	31 (54)/24 (42)	102 (89.47)/3(2.63)	49 (86)/1 (2)
Rahma et al. [23]	2021	RCT	II	United States	Neoadjuvant induction with Folinic Acid, Fluorouracil (5-FU) and Oxaliplatin followed by CRT (Capecitabine) + Pembrolizumab	Neoadjuvant induction with Folinic Acid, Fluorouracil (5-FU) and Oxaliplatin followed by CRT (Capecitabine)	Folinic Acid, Fluorouracil (5-FU) and Oxaliplatin (6 cycles); 3–4 weeks later: Capecitabine (825 mg/m² BID, orally) + LCRT (4500 cGy/25 fractions over 5 weeks + 540 cGy boost in 3 fractions) + Pembrolizumab (200 mg q21d × 6); Surgery 8–12 weeks after the last dose of radiotherapy	Folinic Acid, Fluorouracil (5-FU) and Oxaliplatin (6 cycles); 3–4 weeks later: Capecitabine (825 mg/m² BID, orally) + LCRT (4500 cGy/25 fractions over 5 weeks + 540 cGy boost in 3 fractions); Surgery 8–12 weeks after the last dose of radiotherapy	185 (90/95)	60 (66.7)/66 (69.5)	55.5/56.3^a^	T1/2 = 4 (4.4) T3 = 67 (74.4) T4 = 19 (21.1)	T1/2 = 5 (5.3) T3 = 68 (71.6) T4 = 22 (23.2)	N0 = 20 (22.2) N1 = 35 (38.9) N2 = 35 (38.9)	N0 = 22 (23.2) N1 = 36 (37.9) N2 = 37 (38.9)	25 (27.8)/65 (72.2)	27 (28.4)/68 (71.6)	NR	NR

a: For this study, which reported age distributions in intervals, the median age was estimated using the Group Median Formula

I *Intervention, C* Comparator, *MMR *Mismatch repair, *RCT *Randomized controlled trial, *LCRT *Long-course chemoradiotherapy, *IV *Intravenous, *CRT *Chemoradiotherapy, *NR *Not reported, *SCRT *Short-course radiotherapy, *BID *Twice daily, *Gy *Gray, *cGy *Centigray, *q21d E*very 21 days

### Pathological complete response (pCR) rate

**Fig. 2:**
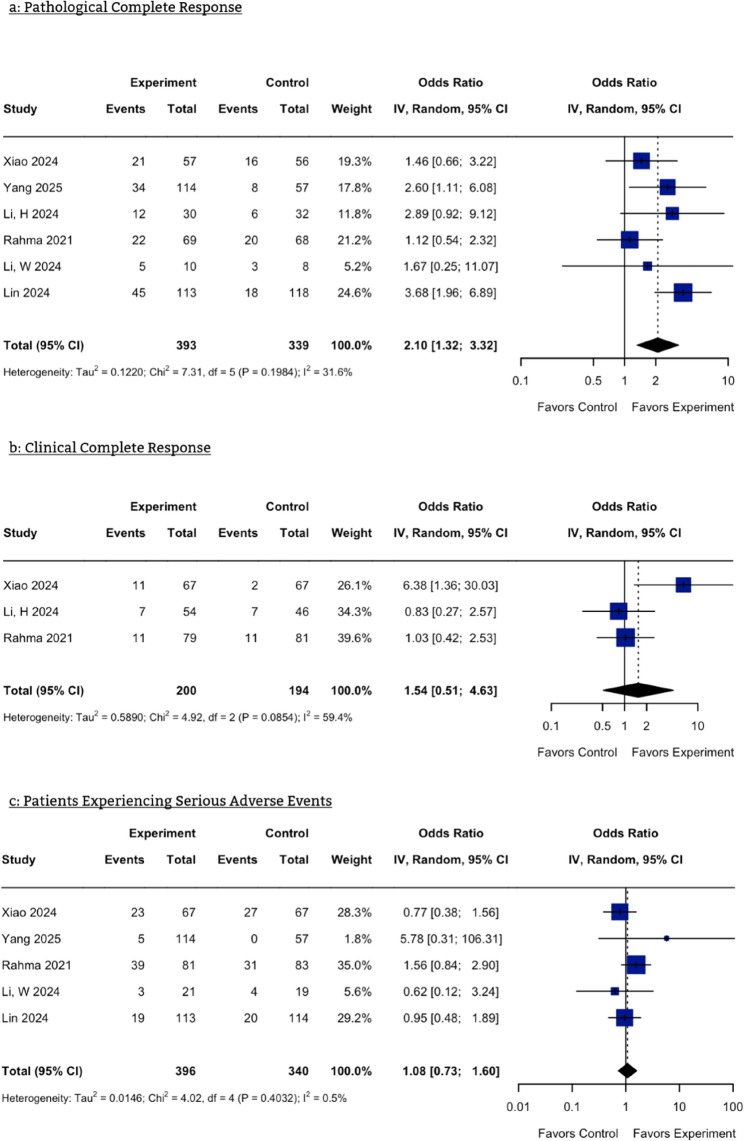
Forest plot of the meta-analysis. **a** Pathological Complete Response **b**: Clinical Complete Response **c**: Serious Adverse Events. CI, Confidence Interval

All of our studies reported pCR rates. This pooled analysis included 732 patients with LARC. A significant difference was found between both arms, with the results favoring neoadjuvant CRT along with a PD-1 inhibitor (OR = 2.10, 95% CI: 1.32–3.32, *p* = 0.001) (Fig. 2a). There was moderate heterogeneity in the results (I² = 31.6%). Upon influence analysis by omitting one study at a time, Rahma et al. [23] and Lin et al. [19] contributed the most to heterogeneity (Figure S 1), and removing them significantly reduced heterogeneity to zero. The results of the Baujat plot and Galbraith plot were consistent with this finding, with all other studies clustering close to the regression line except for these two, which were revealed as outliers (Figures S2 and S3). Results from the DOI plot indicated potential publication bias (LFK index = −1.27) (Figure S4).

### Clinical complete response (cCR) rate

Three of our included studies reported cCR. Analysis of 394 patients with LARC favored neoadjuvant CRT with a PD-1 inhibitor, but the results were statistically non-significant (OR = 1.54, 95% CI: 0.51–4.63, *p* = 0.44) (Fig. 2b). There was substantial heterogeneity present (I² = 59.4%), which, upon influence analysis, was entirely due to the Xiao et al. study [14] (Figure S5), as confirmed by the Baujat plot (Figure S6). Also, the analysis showed potential publication bias, with the LFK index being 2.01 (Figure S7).

### Safety parameters

To compare the safety of both groups, we analyzed the occurrence of grade 3 or higher adverse events. SAEs occurred more frequently when PD-1 inhibitors were combined with CRT, but this difference was not significant (OR = 1.08, 95% CI: 0.73–1.60) (Fig. 2c). There was no significant heterogeneity (I² = 0.5%). The DOI plot gave an LFK index of 1.83, which may indicate potential publication bias (Figure S8).

### Exploratory subgroup analyses

#### Subgroup analysis based on radiotherapy strategies

**Fig. 3:**
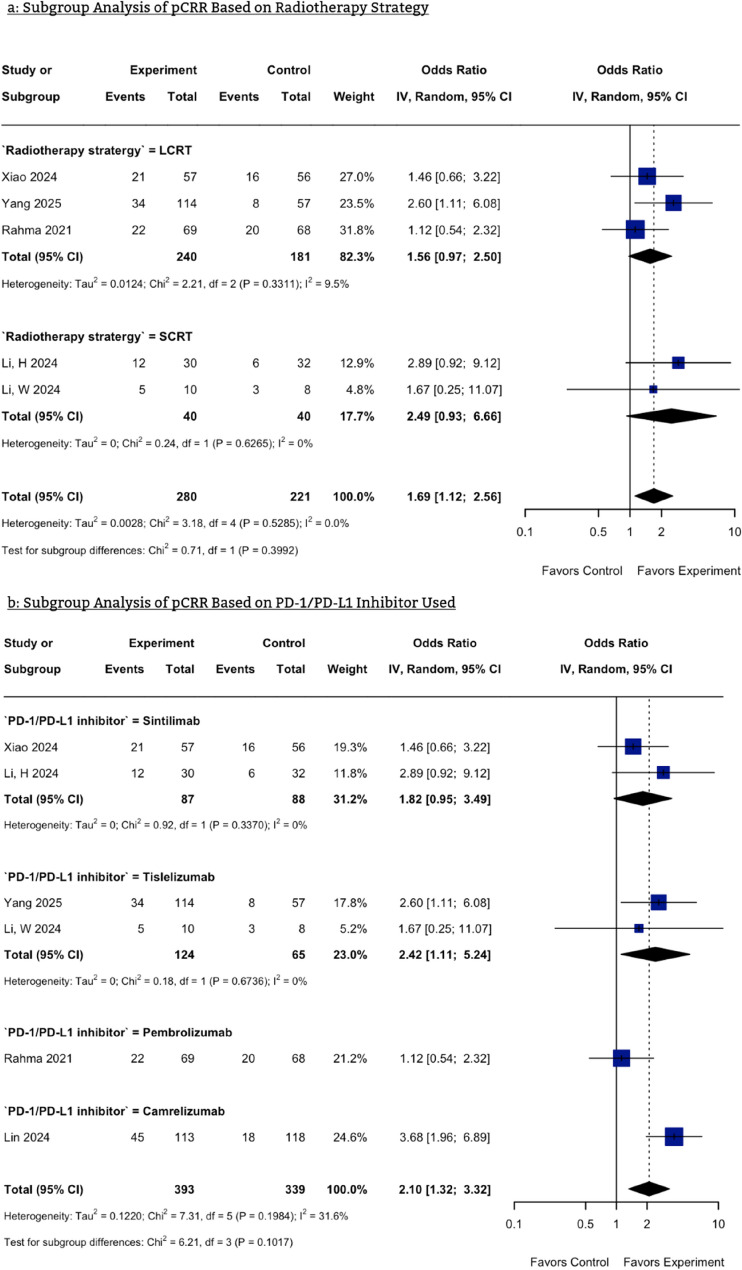
Forest plot of the Subgroup Analyses. **a** Subgroup analysis based on radiotherapy strategies **b**: Subgroup analysis based on PD-1/PD-L1 inhibitor used. pCRR, pathological complete response rate; CI, Confidence Interval; LCRT, Long-Course Radiotherapy b: SCRT, Short-Course Radiotherapy; PD-1/PD-L1, Programmed cell death protein 1/programmed cell death ligand 1

The pathological response rate showed a numerically higher OR with PD-1 inhibitors plus short-course radiotherapy (SCRT) (OR: 2.49, 95% CI: 0.93–6.66) compared to long-course radiotherapy (LCRT) (OR: 1.56, 95% CI: 0.97–2.50). However, the results for both the SCRT and LCRT subgroups were not statistically significant (Fig. 3a). SAEs occurred more frequently in the LCRT subgroup compared to the SCRT subgroup, though both were nonsignificant (OR LCRT: 1.21, 95% CI: 0.63–2.36, OR SCRT: 0.62, 95% CI: 0.12–3.24) (Figure S9a).

#### Subgroup analysis based on PD-1/PD-L1 Inhibitor used

For pathological response rate, Camrelizumab exhibited the highest OR (OR = 3.68, 95% CI: 1.96–6.89), followed by Tislelizumab (OR = 2.42, 95% CI: 1.11–5.24), Sintilimab (OR = 1.82, 95% CI: 0.95–3.49), and Pembrolizumab, which had the lowest OR (OR = 1.12, 95% CI: 0.54–2.32). The results of the Tislelizumab and Camrelizumab subgroups were significant, while the Sintilimab and Pembrolizumab subgroups had non-significant results (Fig. 3b). OR of the Pembrolizumab subgroup was the highest regarding the occurrence of SAEs, followed by Tislelizumab, Camrelizumab, and Sintilimab subgroups, respectively (Figure S9b).

#### Subgroup analysis based on MMR status

**Fig. 4:**
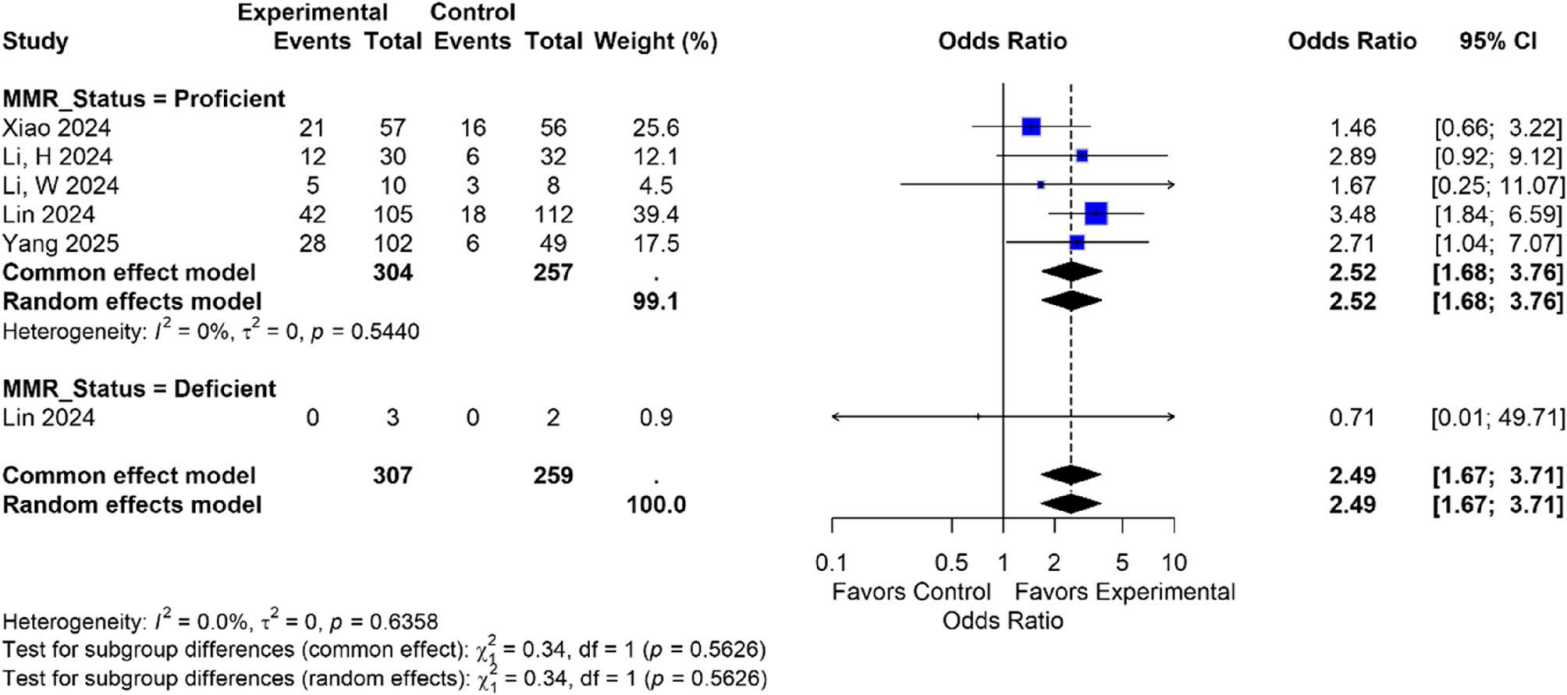
Forest plot of the subgroup analysis based on MMR status. MMR, mismatch repair

For pCR rate, the pMMR subgroup showed a higher OR (OR: 2.52, 95% CI: 1.68–3.76) compared to the dMMR subgroup (OR: 0.71, 95% CI: 0.01–49.71). The result for the pMMR subgroup was statistically significant, while the dMMR subgroup demonstrated a non-significant result, possibly because of the small sample size, and reporting no events in either arm. Overall, the combined OR for both subgroups was 2.49 (95% CI: 1.67–3.71) with no significant heterogeneity between the subgroups (I² = 0.0%) (Fig. 4). Subgroup analysis for SAEs based on MMR status could not be performed because three studies included only pMMR patients [14, 20, 21], two studies [19, 22] did not report SAEs stratified by MMR status, and one study did not report MMR status at all [23].

### Quality assessment of included studies

We assessed the included studies using the Cochrane Risk of Bias assessment for randomized trials (ROB 2) [15]. Of the six included studies, one was at low risk of bias, and five had some concerns. All domains except D1 (randomization process) and D2 (deviations from the intended interventions) showed no concerns (Supplementary Figures S10 and S11). Details of the quality assessment are provided in the supplementary file.

## Discussion

This systematic review and meta-analysis included six RCTs with a total of 861 participants with LARC, of whom 732 were included in the final analysis, and reported a significant increase in pCR rate with neoadjuvant CRT along with PD-1 inhibitors. Among the other outcomes studied, the cCR and safety parameters showed non-significant findings. To the best of our knowledge, this is the first systematic review and meta-analysis evaluating the safety and efficacy of adding PD-1/PD-L1 inhibitors to neoadjuvant CRT in patients with LARC. The existing data have suggested a significant increase in the pCR rate in patients who received CRT with PD-1 inhibitors [14, 19–23], which has been reinforced by our results. The results of Rahma et al.‘s study [23] were non-significant and were the key reason for the heterogeneity observed in the results. Although Rahma et al. reported results that favored the pCR rate of 31.88% in patients who received Pembrolizumab vs. 29.41% in the control group, these results were non-significant (*p* = 0.75). Also, the cCR rate was 13.92% in the group that received Pembrolizumab vs. 13.58% in the control group, with a *p* = 0.95. The reason behind these results could be the lack of completion of all six doses of Pembrolizumab by 44 (54.32%) patients, with 23 (28.39%) receiving fewer than five doses.

Although analysis of cCR in patients with LARC favored neoadjuvant CRT with a PD-1 inhibitor, the results were statistically non-significant. This may be because of several factors: Only three RCTs reported cCR, which could limit statistical power. Substantial heterogeneity was present that, upon influence analysis, was entirely due to the Xiao et al. study [14], which could also weaken the effect estimate. Additionally, as mentioned earlier, in Rahma et al.‘s study [23], over half the patients failed to complete all six doses of Pembrolizumab, potentially compromising immunotherapeutic efficacy. Differences in imaging modalities, cCR definitions, and timing of response assessment across studies can cause further inconsistency.

This meta-analysis demonstrated that SAEs occurred more frequently when PD-1 inhibitors were combined with CRT, but this difference was not significant. Li H et al. [21] showed a higher rate of grade 3–5 toxicities in the immunotherapy plus CRT group compared to CRT alone, with thrombocytopenia most common in both groups. Xiao et al. [14] found myelosuppression as the most common grade 3–4 toxicity during neoadjuvant treatment. In Li W et al.‘s study [20], grade 3 treatment-related adverse events (TRAEs) were 14.3% in the Tislelizumab plus CRT group and 21.5% in the CRT group, and one immune-related grade 3 myositis was reported that required treatment interruption. Yang et al. [22] observed grade 3–4 neoadjuvant-TRAEs, with the most common related to gastrointestinal and skin toxicities. Lin et al. [19] noted hematologic AEs as the most common ones across groups. Also, Rahma et al. [23] found more SAEs with Pembrolizumab (48.2% vs. 37.3%). Overall, while some increase in SAEs was observed, it was not statistically significant, and most events were manageable, supporting the tolerability of PD-1 inhibitors in the neoadjuvant setting.

The current treatment options available for patients with LARC are a long/short course of radiotherapy and CAPOX (Capecitabine and Oxaliplatin) or FOLFOX (Folinic Acid, Fluorouracil [5-FU], Oxaliplatin), scheduled to receive radical surgery. The discovery of neoadjuvant immunotherapy in clinical trials has led to an exciting breakthrough in response rates compared to previously used neoadjuvant CRT [11, 24]. Immune checkpoint inhibitors (ICIs) improve the immune system’s capacity to recognize and destroy cancer cells. Bypassing the immune response is a key mechanism of cancer development and progression [25]. Cancer cells survive by constantly altering the immune checkpoints responsible for the activation and progression of a normal immune response. Of these, the two pathways, mainly cytotoxic T lymphocyte antigen 4 (CTLA-4) and PD-1, are highly involved [26, 27]. PD-1 is a transmembrane molecule with two ligands (PD-L1 and PD-L2) that binds and inhibits T cell proliferation and differentiation, blocking signal transduction of various cytokines, causing tumor progression and metastasis [28, 29]. In the tumor microenvironment, the constantly rapidly dividing cells express a variety of neoantigens to help evade the immune response and escape apoptosis. PD-L1 is a key immune regulator; it is an apoptotic protein that binds with the PD-1 on T lymphocytes, causing apoptosis, inactivity, T cell exhaustion, accumulation of T regulatory cells, and development of immune tolerance of T cells [30].

The two hypotheses associated with the upregulation of PD-1 in tumor immune evasion are as follows: The first hypothesis implies that the change in genome caused by the tumor cells upregulates several pathways, including Akt and STAT. *PTEN* gene deletion in the tumor cells increases the PI3K/AKT pathway, upregulating the expression of PD-1/PD-L1 in tumor cells [31, 32]. The second hypothesis implies the tumor cells’ protective response from being destroyed by the immune system. The tumor cells release interferon-γ (IFN-γ) in response to CD4, Type 1 T helper (Th1), and activated T cells, which upregulate the production of PD-L1, shutting the T cells down [33]. This signifies the key role of ICIs in LARC and the statistically significant data supporting the results of their emerging use in treatment therapies.

Recent studies have demonstrated how neoadjuvant CRT synergizes with PD-1 blockade. CRT has been shown to induce immunogenic cell death (ICD), releasing tumor-associated antigens and damage-associated molecular patterns (DAMPs) that promote antigen presentation and T-cell activation [34]. However, as mentioned earlier, this immune activation also induces PD-L1 expression in the tumor microenvironment, leading to suppressed T-cell function. Anti-PD-1 therapies inhibit this resistance, which results in sustained T-cell activity and increased anti-tumor immune responses during CRT treatment [35]. In colorectal tumors, CRT has also been correlated with elevated CD8 + T-cell infiltration and IFN-γ signatures [36, 37]. As mentioned before, tumor genomic alterations, such as *PTEN* loss and PI3K/AKT pathway activation, can elevate PD-L1 expression, which can lead to increased susceptibility to ICIs. Thus, integrating PD-1 inhibitors with CRT in the neoadjuvant setting may potentiate both direct cytotoxic and immune-mediated tumor eradication.

This meta-analysis explores broad approaches, including CRT combined with immunotherapy, which has been shown to enhance the pCR rate in tumors. A meta-analysis by Turri et al. focused solely on CRT regimens for LARC [6], evaluating variations within TNT and excluding immunotherapy. It identified CRT with consolidation chemotherapy as the most effective, achieving a higher pCR rate compared to standard CRT. While Turri et al. demonstrated CRT’s efficacy in standard treatment settings, the discussion section of this study highlighted the added potential of immunotherapy to improve outcomes in molecularly defined tumor subsets.

Recent trials integrating PD-1/PD-L1 blockade into neoadjuvant CRT regimens for LARC have demonstrated promising results, albeit with varying levels of efficacy and safety. Studies such as the TORCH trial [38], STELLAR II [21], and others have consistently indicated that the addition of immunotherapeutic agents like PD-1 inhibitors enhances CR rates, particularly in patients with pMMR or microsatellite-stable (MSS) LARC. For instance, the STELLAR II trial reported a CR rate of 40.4% in its immunotherapy-integrated TNT group compared to 31.0% in the control [21]. Similarly, the TORCH trial reported an improved CR rate across the treatment group [38].

These findings align with those reported by Xiao et al., who observed a significant increase in CR rates when Sintilimab was added to neoadjuvant CRT [14]. Likewise, Yang et al.‘s trial showed that the addition of PD-1 inhibitors such as Tislelizumab raised the pCR rate when combined with CRT [22].

This meta-analysis highlights the variability in clinical and pCR rates across trials integrating PD-1 blockade. The study by Cercek et al. reported a 100% cCR rate in patients with dMMR rectal cancers using Dostarlimab monotherapy as immunotherapy alone [11]. However, this meta-analysis integrates heterogeneous studies with varying immunotherapy combinations, treatment protocols, and inclusion criteria. The strict focus on dMMR and the inclusion of non-metastatic tumors in the Cercek et al. study [11] may explain its uniformly high response rate. The 100% response in the Cercek et al. study underscores the significance of dMMR status, which induces a hypermutated tumor phenotype and heightened immunogenicity due to increased neoantigen load. This contrasts with the diverse tumor profiles analyzed in this meta-analysis.

MMR status plays a crucial role in determining the immune response and therapeutic outcomes in colorectal cancer. Microsatellite instability-high (MSI-high) tumors, associated with dMMR, often exhibit increased immune infiltration and a higher neoantigen load, making them more responsive to immunotherapy, such as PD-1 inhibitors. In contrast, pMMR or MSS tumors typically show lower immunogenicity and reduced response rates to such treatments [39]​. Additionally, metastatic colorectal cancers generally display a lower response to immunotherapy compared to localized tumors, which is attributed to a more immunosuppressive microenvironment at metastatic sites, characterized by factors such as altered immune cell composition, reduced T-cell infiltration, and the presence of *Fusobacterium nucleatum*, which negatively impacts adaptive immune responses [39]​. These findings highlight the importance of tailoring treatment strategies based on MMR status and tumor localization.

In this review, the subgroup analysis based on radiotherapy strategies showed that PD-1 inhibitors combined with CRT were correlated with a numerically higher pathological response rate in the SCRT group compared to the LCRT group. However, neither subgroup reached statistical significance, preventing definitive conclusions about the superiority of one approach over the other. Although this result was also not statistically significant in the meta-analysis, the analysis showed that SAEs were more common in the LCRT subgroup, demonstrating the need for more studies to identify the optimal radiotherapy strategies. Additionally, the results indicated that, among the PD-1 inhibitors, Camrelizumab and Tislelizumab had the most significant impact on pathological response rates, while Sintilimab and Pembrolizumab did not show statistically significant effects. Moreover, Pembrolizumab was associated with the highest rate of SAEs. These findings indicate the need for further investigation to identify the safest and most effective PD-1 inhibitors for patient treatment. Additionally, the subgroup analysis based on MMR status demonstrates critical insights into the differential efficacy of PD-1/PD-L1 inhibitors in patients with LARC. The substantial benefit observed in the pMMR subgroup is possibly due to the larger sample size and higher event rates in this population, suggesting that the synergistic effects of PD-1 inhibitors with CRT may be more prominent in pMMR tumors, despite their lower neoantigen load compared to dMMR tumors. Furthermore, the absence of pCR in the dMMR subgroup highlights a limitation in assessing the efficacy of PD-1/PD-L1 inhibitors in this group, potentially due to the small number of dMMR patients and insufficient reporting of MMR-stratified outcomes. In addition, the inability to evaluate SAEs by MMR status underscores the need for standardized reporting in future studies to better understand the safety profile of these therapies in molecular subgroups.

The increased CR rates observed across trials suggest that combining immunotherapy with CRT could represent a significant step forward for LARC management. However, results such as those from the Li, W et al. trial, which found no significant improvement in NAR scores despite immunotherapy use, highlight the need for continued refinement in patient selection and therapeutic protocols [20]. Overall, this meta-analysis reinforces the notion that integrating PD-1 inhibitors can offer meaningful benefits, though their precise role remains contingent on tumor biology and patient factors.

### Strengths and limitations

This review synthesizes data from diverse, high-quality studies, offering a comprehensive evaluation of PD-1 blockade efficacy in LARC. Its emphasis on phase II/III RCTs enhances the robustness of its findings. By focusing on both pCR and cCR rates and adverse event profiles, the analysis provides a balanced view of clinical benefits and potential risks. Also, performing subgroup analysis based on MMR status strengthens the study by showing the effect of tumor biology on treatment outcomes, especially the differential response between pMMR and dMMR tumors.

Despite its strengths, there are limitations to this analysis. The relatively small available sample size may limit the statistical power and strength of our findings. In addition, variability in treatment protocols, patient inclusion criteria (e.g., pMMR vs. dMMR stratification), and outcome definitions across studies may introduce heterogeneity. The limited number of dMMR patients and the lack of stratified reporting for outcomes such as SAEs by MMR status in the included studies limit the ability to draw definitive conclusions about the role of MMR status in both the efficacy and safety of the usage of PD-1/PD-L1 inhibitors. Furthermore, longer-term endpoints such as disease-free survival (DFS) and OS remain underreported in many trials, limiting conclusions on long-term efficacy. Lastly, the included trials predominantly represent patients from certain geographic and demographic backgrounds, potentially restricting generalizability.

### Implications for practice, policy, and future research

The integration of PD-1 inhibitors into the therapeutic arsenal for LARC appears to enhance pCR rates significantly, offering a potential pathway for improved tumor downstaging and organ preservation strategies [14, 22]. While our analysis was limited to short-term endpoints such as pCR and cCR, there is evidence that achieving a higher pCR correlates with improved long-term outcomes in patients with LARC [40]. Thus, the higher pCR rates observed with the addition of PD-1 inhibitors in our meta-analysis may translate into improved survival outcomes, although more trials with longer follow-up durations are needed to confirm these findings. Also, clinicians should consider tumor molecular profiles, such as pMMR/MSS status, when designing treatment regimens.

Regulatory bodies should prioritize the inclusion of PD-1 inhibitors in updated treatment guidelines for LARC, particularly as part of TNT protocols [5]. However, a broader availability of diagnostic tools for MSI/MMR testing is essential to optimize patient stratification [41].

Several areas warrant further exploration. Phase III trials with larger cohorts and diverse populations are needed to validate these findings. Investigations into biomarkers, such as PD-L1 expression and immune signatures, could refine patient selection criteria [42]. Future studies should prioritize the inclusion of dMMR patients and the stratified reporting of outcomes, including SAEs by MMR status, to better understand the differential effects of PD-1/PD-L1 inhibitors in these subgroups. Moreover, exploring novel combinations of CRT, immunotherapy, and targeted agents may uncover synergistic strategies to further enhance outcomes.

## Conclusion

This systematic review and meta-analysis highlights the therapeutic benefit of combining chemotherapy and radiotherapy with PD-1 inhibitors in the neoadjuvant setting by showing superior results for pCR. More clinical trials with longer follow-up durations are needed to confirm these findings and incorporate the use of PD-1/PD-L1 inhibitors alongside chemotherapy and radiotherapy in clinical guidelines.

## Supplementary Information

Supplementary Material 1

## Data Availability

All data generated or analyzed during this study are included in this published article and its supplementary information file.

## Abbreviations

LARC: Locally advanced rectal cancer
MRI: Magnetic Resonance Imaging
MMR: Mismatch repair
TNT: Total neoadjuvant therapy
CRT: Chemoradiotherapy
OS: Overall survival
PD-1: Programmed cell death protein 1
PD-L1: Programmed cell death ligand 1
CR: Complete response
SAEs: Serious adverse events
RCTs: Randomized controlled trials
ASCO: American Society of Clinical Oncology
Pcr: Pathological complete response
cCR: Clinical complete response
pMMR: Proficient MMR
dMMR: Deficient MMR
ROB 2: Risk of Bias 2
Ors: Odds ratios
Cis: Confidence intervals
SCRT: Short-course radiotherapy
LCRT: Long-course radiotherapy
CAPOX: Capecitabine and Oxaliplatin
FOLFOX: Folinic Acid, Fluorouracil (5-FU), Oxaliplatin
ICIs: Immune checkpoint inhibitors
CTLA-4: Cytotoxic T lymphocyte antigen 4
IFN-γ: Interferon-γ
Th1: Type 1 T helper
MSS: Microsatellite-stable
MSI-high: Microsatellite instability-high
DFS: Disease-free survival
