# Supplementary material for: Neoadjuvant chemoradiotherapy with or without PD-1/PD-L1 inhibitors in locally advanced rectal cancer: a systematic review and meta-analysis
Source: BMC Cancer. 2025 Jul 1;25:1084. doi: 10.1186/s12885-025-14482-5 (PMC12211167; doi:10.1186/s12885-025-14482-5)
Supplement: Supplementary file 1 — Supplementary Material 1 [file 12885_2025_14482_MOESM1_ESM.docx]

**Supplemental Material**

Neoadjuvant Chemoradiotherapy With or Without PD-1/PD-L1 Inhibitors in Locally Advanced Rectal Cancer: A Systematic Review and Meta-Analysis

This supplemental material is provided by the authors for a better understanding of their work

**Supplementary Table S1:** Prisma Checklist

**Supplementary Table S2:** Detailed Search Strategy of Each Database

**Supplementary Figure S1:** Influence analysis of pathological complete response (pCR) rate.

**Supplementary Figure S2:** Baujat plot of pathological complete response (pCR) rate.

**Supplementary Figure S3:** Galbraith plot of pathological complete response (pCR) rate.

**Supplementary Figure S4:** DOI plot of pathological complete response (pCR) rate.

**Supplementary Figure S5:** Influence analysis of clinical complete response (cCR) rate.

**Supplementary Figure S6:** Baujat plot of clinical complete response (cCR) rate.

**Supplementary Figure S7:** DOI plot of clinical complete response (cCRR) rate.

**Supplementary Figure S8:** DOI plot of serious adverse events (SAEs).

**Supplementary Figure S9:** Subgroup analysis of SAEs based on a: Type of radiotherapy strategy b: PD-1/PD-L1 inhibitor used.

**Supplementary Figure S10:** Risk-of-bias summary illustrating review authors' judgments for each risk-of-bias item across all included studies.

**Supplementary Figure S11:** Risk-of-bias graph illustrating review authors' judgments for each risk-of-bias item, presented as percentages across all included studies.

**Supplementary Table S1:** Prisma Checklist

| **Section and Topic** | **Item #** | **Checklist item** | **Location where item is reported** |
| --- | --- | --- | --- |
| **TITLE** | | |  |
| Title | 1 | Identify the report as a systematic review. | Line: 1-3 |
| **ABSTRACT** | | |  |
| Abstract | 2 | See the PRISMA 2020 for Abstracts checklist. | Line 37-69 |
| **INTRODUCTION** | | |  |
| Rationale | 3 | Describe the rationale for the review in the context of existing knowledge. | Line: 123-135 |
| Objectives | 4 | Provide an explicit statement of the objective(s) or question(s) the review addresses. | Line: 135-138 |
| **METHODS** | | |  |
| Eligibility criteria | 5 | Specify the inclusion and exclusion criteria for the review and how studies were grouped for the syntheses. | Line: 155-172 |
| Information sources | 6 | Specify all databases, registers, websites, organisations, reference lists and other sources searched or consulted to identify studies. Specify the date when each source was last searched or consulted. | Line: 145-154 |
| Search strategy | 7 | Present the full search strategies for all databases, registers and websites, including any filters and limits used. | Supplementary Table 2 |
| Selection process | 8 | Specify the methods used to decide whether a study met the inclusion criteria of the review, including how many reviewers screened each record and each report retrieved, whether they worked independently, and if applicable, details of automation tools used in the process. | Line: 159-164 |
| Data collection process | 9 | Specify the methods used to collect data from reports, including how many reviewers collected data from each report, whether they worked independently, any processes for obtaining or confirming data from study investigators, and if applicable, details of automation tools used in the process. | Line: 183-189 |
| Data items | 10a | List and define all outcomes for which data were sought. Specify whether all results that were compatible with each outcome domain in each study were sought (e.g. for all measures, time points, analyses), and if not, the methods used to decide which results to collect. | Line: 180-181 |
|  | 10b | List and define all other variables for which data were sought (e.g. participant and intervention characteristics, funding sources). Describe any assumptions made about any missing or unclear information. | Line: 174-183 |
| Study risk of bias assessment | 11 | Specify the methods used to assess risk of bias in the included studies, including details of the tool(s) used, how many reviewers assessed each study and whether they worked independently, and if applicable, details of automation tools used in the process. | Line: 190-203 |
| Effect measures | 12 | Specify for each outcome the effect measure(s) (e.g. risk ratio, mean difference) used in the synthesis or presentation of results. | Line: 205-207 |
| Synthesis methods | 13a | Describe the processes used to decide which studies were eligible for each synthesis (e.g. tabulating the study intervention characteristics and comparing against the planned groups for each synthesis (item #5)). | Line: 159-164 |
|  | 13b | Describe any methods required to prepare the data for presentation or synthesis, such as handling of missing summary statistics, or data conversions. | Line: 185-189 |
|  | 13c | Describe any methods used to tabulate or visually display results of individual studies and syntheses. | Line:199-203, 207, 213-216 |
|  | 13d | Describe any methods used to synthesize results and provide a rationale for the choice(s). If meta-analysis was performed, describe the model(s), method(s) to identify the presence and extent of statistical heterogeneity, and software package(s) used. | Line: 204-220 |
|  | 13e | Describe any methods used to explore possible causes of heterogeneity among study results (e.g. subgroup analysis, meta-regression). | Line: 209-219 |
|  | 13f | Describe any sensitivity analyses conducted to assess robustness of the synthesized results. | Line: 207-209 |
| Reporting bias assessment | 14 | Describe any methods used to assess risk of bias due to missing results in a synthesis (arising from reporting biases). | Line: 199-203 |
| Certainty assessment | 15 | Describe any methods used to assess certainty (or confidence) in the body of evidence for an outcome. | - |
| **RESULTS** | | |  |
| Study selection | 16a | Describe the results of the search and selection process, from the number of records identified in the search to the number of studies included in the review, ideally using a flow diagram. | Line: 223-229, Figure 1 |
|  | 16b | Cite studies that might appear to meet the inclusion criteria, but which were excluded, and explain why they were excluded. | - |
| Study characteristics | 17 | Cite each included study and present its characteristics. | Table 1 |
| Risk of bias in studies | 18 | Present assessments of risk of bias for each included study. | Figure S10 |
| Results of individual studies | 19 | For all outcomes, present, for each study: (a) summary statistics for each group (where appropriate) and (b) an effect estimate and its precision (e.g. confidence/credible interval), ideally using structured tables or plots. | Figures 2, 3, S1-S9 |
| Results of syntheses | 20a | For each synthesis, briefly summarise the characteristics and risk of bias among contributing studies. | Line: 240-286 |
|  | 20b | Present results of all statistical syntheses conducted. If meta-analysis was done, present for each the summary estimate and its precision (e.g. confidence/credible interval) and measures of statistical heterogeneity. If comparing groups, describe the direction of the effect. | Line: 240-280 |
|  | 20c | Present results of all investigations of possible causes of heterogeneity among study results. | Line: 240-280 |
|  | 20d | Present results of all sensitivity analyses conducted to assess the robustness of the synthesized results. | Line: 240-257 |
| Reporting biases | 21 | Present assessments of risk of bias due to missing results (arising from reporting biases) for each synthesis assessed. | Figure S10 |
| Certainty of evidence | 22 | Present assessments of certainty (or confidence) in the body of evidence for each outcome assessed. | Figures 2, 3, S1, S5, S9 |
| **DISCUSSION** | | |  |
| Discussion | 23a | Provide a general interpretation of the results in the context of other evidence. | Line: 294-371 |
|  | 23b | Discuss any limitations of the evidence included in the review. | Line: 296-303 |
|  | 23c | Discuss any limitations of the review processes used. | Line: 396-401 |
|  | 23d | Discuss implications of the results for practice, policy, and future research. | Line: 402-414 |
| **OTHER INFORMATION** | | |  |
| Registration and protocol | 24a | Provide registration information for the review, including register name and registration number, or state that the review was not registered. | Line: 141-144 |
|  | 24b | Indicate where the review protocol can be accessed, or state that a protocol was not prepared. | PROSPERO |
|  | 24c | Describe and explain any amendments to information provided at registration or in the protocol. | - |
| Support | 25 | Describe sources of financial or non-financial support for the review, and the role of the funders or sponsors in the review. | - |
| Competing interests | 26 | Declare any competing interests of review authors. | Line: 438-446 |
| Availability of data, code and other materials | 27 | Report which of the following are publicly available and where they can be found: template data collection forms; data extracted from included studies; data used for all analyses; analytic code; any other materials used in the review. | - |

**Supplementary Table S2:** Detailed Search Strategy of Each Database

| **Data Base** | **Search String** | **Results** |
| --- | --- | --- |
| PubMed | ((((((((((((((("Rectal Neoplasms"[Mesh]) OR (Locally advanced rectal cancer)) OR (LARC)) OR (Rectal Neoplasm)) OR (Rectum Neoplasms)) OR (Rectum Neoplasm)) OR (Rectal Tumors)) OR (Rectal Tumor)) OR (Cancer of Rectum)) OR (Rectum Cancers)) OR (Cancer of the Rectum)) OR (Rectal Cancer)) OR (Rectal Cancers)) OR (Rectum Cancer))  AND  (((((((((((((((((((((((((((("Neoadjuvant Therapy"[Mesh]) OR (Neoadjuvant Therapies)) OR (Neoadjuvant Treatment)) OR (Neoadjuvant Treatments)) OR (Neoadjuvant Chemotherapy)) OR (Neoadjuvant Chemotherapies)) OR (Neoadjuvant Chemotherapy Treatment)) OR (Neoadjuvant Chemotherapy Treatments)) OR (Neoadjuvant Chemoradiotherapy)) OR (Neoadjuvant Chemoradiotherapies)) OR (Neoadjuvant Chemoradiation)) OR (Neoadjuvant Chemoradiations)) OR (Neoadjuvant Chemoradiation Therapy)) OR (Neoadjuvant Chemoradiation Therapies)) OR (Neoadjuvant Chemoradiation Treatment)) OR (Neoadjuvant Chemoradiation Treatments)) OR (Neoadjuvant Systemic Therapy)) OR (Neoadjuvant Systemic Therapies)) OR (Neoadjuvant Systemic Treatment)) OR (Neoadjuvant Systemic Treatments)) OR (Neoadjuvant Radiotherapy)) OR (Neoadjuvant Radiotherapies)) OR (Neoadjuvant Radiation)) OR (Neoadjuvant Radiations)) OR (Neoadjuvant Radiation Treatment)) OR (Neoadjuvant Radiation Treatments)) OR (Neoadjuvant Radiation Therapy)) OR (Neoadjuvant Radiation Therapies)))  AND  (((((((((((((((((((((((((((((((((((((((("Nivolumab"[Mesh]) OR (MDX-1106)) OR (MDX1106)) OR (MDX 1106)) OR (Opdivo)) OR (BMS-936558)) OR (BMS936558)) OR (BMS 936558)) OR (ONO-4538)) OR (ONO4538)) OR (ONO 4538)) OR (cemiplimab)) OR (REGN2810)) OR (PD-1 Inhibitors)) OR (PD 1 Inhibitors)) OR (Programmed Cell Death Protein 1 Inhibitor)) OR (Programmed Cell Death Protein 1 Inhibitors)) OR (PD-1 Inhibitor)) OR (PD 1 Inhibitor)) OR (PD-L1 Inhibitors)) OR (PD L1 Inhibitors)) OR (Programmed Death-Ligand 1 Inhibitors)) OR (Programmed Death Ligand 1 Inhibitors)) OR (PD-L1 Inhibitor)) OR (PD L1 Inhibitor)) OR (PD-1-PD-L1 Blockade)) OR (PD 1 PD L1 Blockade)) OR (pidilizumab)) OR (CT-011)) OR (CT 011)) OR (toripalimab)) OR (dostarlimab)) OR (dostarlimab-gxly)) OR (Jemperli)) OR (GSK4057190)) OR (TSR-042)) OR (sintilimab)) OR (IBI 308)) OR (IBI308)) OR (IBI-308)) | 119 results (11/10/2024) |
| Cochrane Central Library | ("Rectal Neoplasms" OR "Locally advanced rectal cancer" OR LARC OR "Rectal Neoplasm" OR "Rectum Neoplasms" OR "Rectum Neoplasm" OR "Rectal Tumors" OR "Rectal Tumor" OR "Cancer of Rectum" OR "Rectum Cancers" OR "Cancer of the Rectum" OR "Rectal Cancer" OR "Rectal Cancers" OR "Rectum Cancer")  AND  ("Neoadjuvant Therapy" OR "Neoadjuvant Therapies" OR "Neoadjuvant Treatment" OR "Neoadjuvant Treatments" OR "Neoadjuvant Chemotherapy" OR "Neoadjuvant Chemotherapies" OR "Neoadjuvant Chemotherapy Treatment" OR "Neoadjuvant Chemotherapy Treatments" OR "Neoadjuvant Chemoradiotherapy" OR "Neoadjuvant Chemoradiotherapies" OR "Neoadjuvant Chemoradiation" OR "Neoadjuvant Chemoradiations" OR "Neoadjuvant Chemoradiation Therapy" OR "Neoadjuvant Chemoradiation Therapies" OR "Neoadjuvant Chemoradiation Treatment" OR "Neoadjuvant Chemoradiation Treatments" OR "Neoadjuvant Systemic Therapy" OR "Neoadjuvant Systemic Therapies" OR "Neoadjuvant Systemic Treatment" OR "Neoadjuvant Systemic Treatments" OR "Neoadjuvant Radiotherapy" OR "Neoadjuvant Radiotherapies" OR "Neoadjuvant Radiation" OR "Neoadjuvant Radiations" OR "Neoadjuvant Radiation Treatment" OR "Neoadjuvant Radiation Treatments" OR "Neoadjuvant Radiation Therapy" OR "Neoadjuvant Radiation Therapies")  AND  ("Nivolumab" OR "MDX-1106" OR MDX1106 OR "MDX 1106" OR "Opdivo" OR "BMS-936558" OR BMS936558 OR "BMS 936558" OR "ONO-4538" OR ONO4538 OR "ONO 4538" OR cemiplimab OR REGN2810 OR "PD-1 Inhibitors" OR "PD 1 Inhibitors" OR "Programmed Cell Death Protein 1 Inhibitor" OR "Programmed Cell Death Protein 1 Inhibitors" OR "PD-1 Inhibitor" OR "PD 1 Inhibitor" OR "PD-L1 Inhibitors" OR "PD L1 Inhibitors" OR "Programmed Death-Ligand 1 Inhibitors" OR "Programmed Death Ligand 1 Inhibitors" OR "PD-L1 Inhibitor" OR "PD L1 Inhibitor" OR "PD-1-PD-L1 Blockade" OR "PD 1 PD L1 Blockade" OR pidilizumab OR "CT-011" OR "CT 011" OR toripalimab OR dostarlimab OR "dostarlimab-gxly" OR Jemperli OR GSK4057190 OR "TSR-042" OR sintilimab OR "IBI 308" OR IBI308 OR "IBI-308") | 27 results (11/10/2024) |
| Embase | ('rectal neoplasms'/exp OR 'locally advanced rectal cancer' OR 'larc' OR 'rectal neoplasm' OR 'rectum neoplasms' OR 'rectum neoplasm' OR 'rectal tumors' OR 'rectal tumor' OR 'cancer of rectum' OR 'rectum cancers' OR 'cancer of the rectum' OR 'rectal cancer' OR 'rectal cancers' OR 'rectum cancer')  AND  ('neoadjuvant therapy'/exp OR 'neoadjuvant therapies' OR 'neoadjuvant treatment' OR 'neoadjuvant treatments' OR 'neoadjuvant chemotherapy' OR 'neoadjuvant chemotherapies' OR 'neoadjuvant chemotherapy treatment' OR 'neoadjuvant chemotherapy treatments' OR 'neoadjuvant chemoradiotherapy' OR 'neoadjuvant chemoradiotherapies' OR 'neoadjuvant chemoradiation' OR 'neoadjuvant chemoradiations' OR 'neoadjuvant chemoradiation therapy' OR 'neoadjuvant chemoradiation therapies' OR 'neoadjuvant chemoradiation treatment' OR 'neoadjuvant chemoradiation treatments' OR 'neoadjuvant systemic therapy' OR 'neoadjuvant systemic therapies' OR 'neoadjuvant systemic treatment' OR 'neoadjuvant systemic treatments' OR 'neoadjuvant radiotherapy' OR 'neoadjuvant radiotherapies' OR 'neoadjuvant radiation' OR 'neoadjuvant radiations' OR 'neoadjuvant radiation treatment' OR 'neoadjuvant radiation treatments' OR 'neoadjuvant radiation therapy' OR 'neoadjuvant radiation therapies')  AND  ('nivolumab'/exp OR 'mdx-1106' OR 'mdx1106' OR 'mdx 1106' OR 'opdivo' OR 'bms-936558' OR 'bms936558' OR 'bms 936558' OR 'ono-4538' OR 'ono4538' OR 'ono 4538' OR 'cemiplimab' OR 'regn2810' OR 'pd-1 inhibitors' OR 'pd 1 inhibitors' OR 'programmed cell death protein 1 inhibitor' OR 'programmed cell death protein 1 inhibitors' OR 'pd-1 inhibitor' OR 'pd 1 inhibitor' OR 'pd-l1 inhibitors' OR 'pd l1 inhibitors' OR 'programmed death-ligand 1 inhibitors' OR 'programmed death ligand 1 inhibitors' OR 'pd-l1 inhibitor' OR 'pd l1 inhibitor' OR 'pd-1-pd-l1 blockade' OR 'pd 1 pd l1 blockade' OR 'pidilizumab' OR 'ct-011' OR 'ct 011' OR 'toripalimab' OR 'dostarlimab' OR 'dostarlimab-gxly' OR 'jemperli' OR 'gsk4057190' OR 'tsr-042' OR 'sintilimab' OR 'ibi 308' OR 'ibi308' OR 'ibi-308') | 156 results  10/11/2024 |
| Clinicaltrials.gov | (("Rectal Neoplasms" OR "Locally advanced rectal cancer" OR LARC OR "Rectal Cancer")  AND  ("Neoadjuvant Therapy" OR "Neoadjuvant Chemotherapy" OR "Neoadjuvant Chemoradiotherapy" OR  "Neoadjuvant Radiation Therapy")  AND  ("Nivolumab" OR "PD-1 Inhibitor" OR "PD-L1 Inhibitor" OR "Opdivo" OR "Cemiplimab" OR  "Dostarlimab" OR "Toripalimab" OR "Sintilimab" OR "PD-1/PD-L1 Blockade")) | 19 results  10/11/2024 |


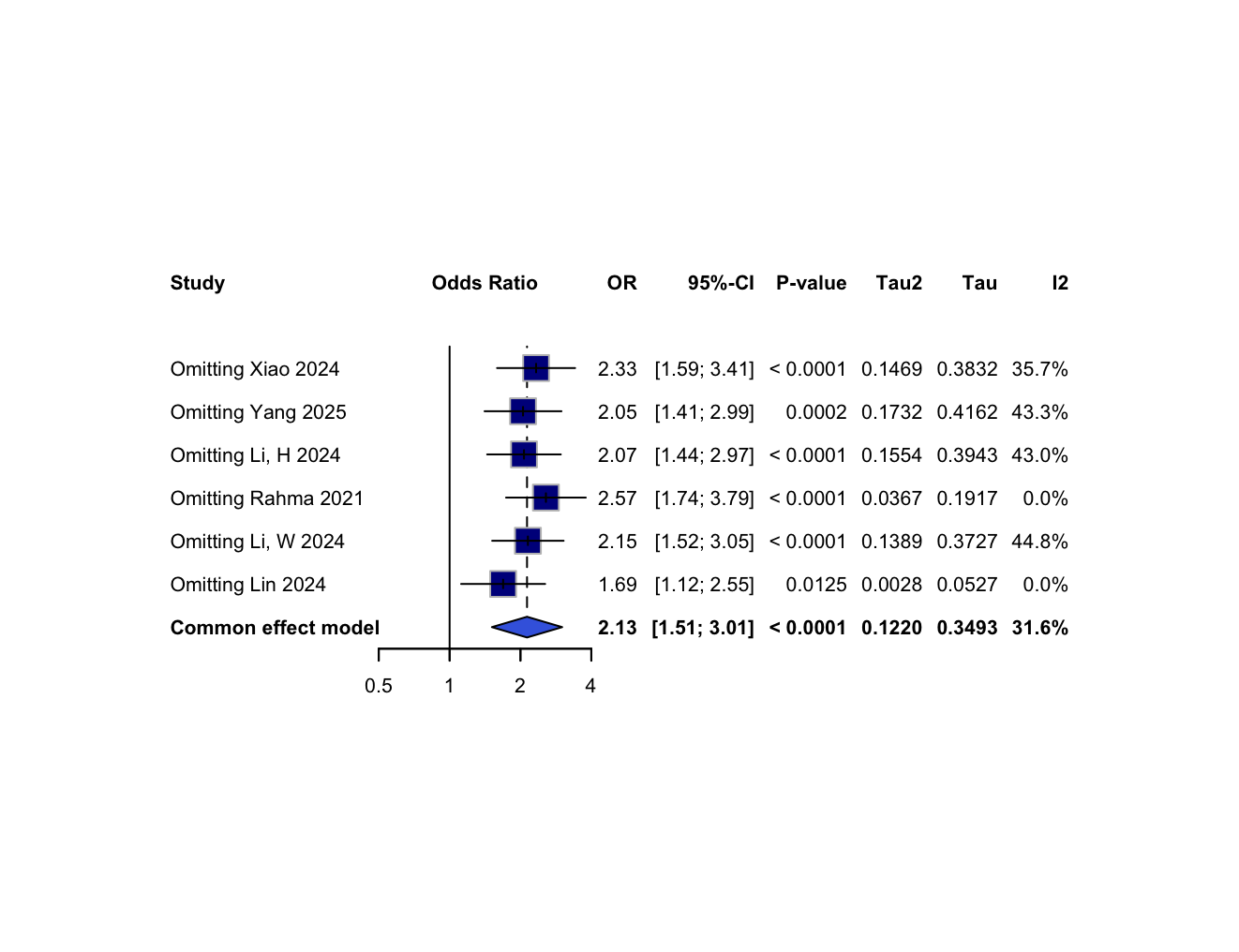


**Supplementary Figure S1:** Influence analysis of pathological complete response (pCR) rate.


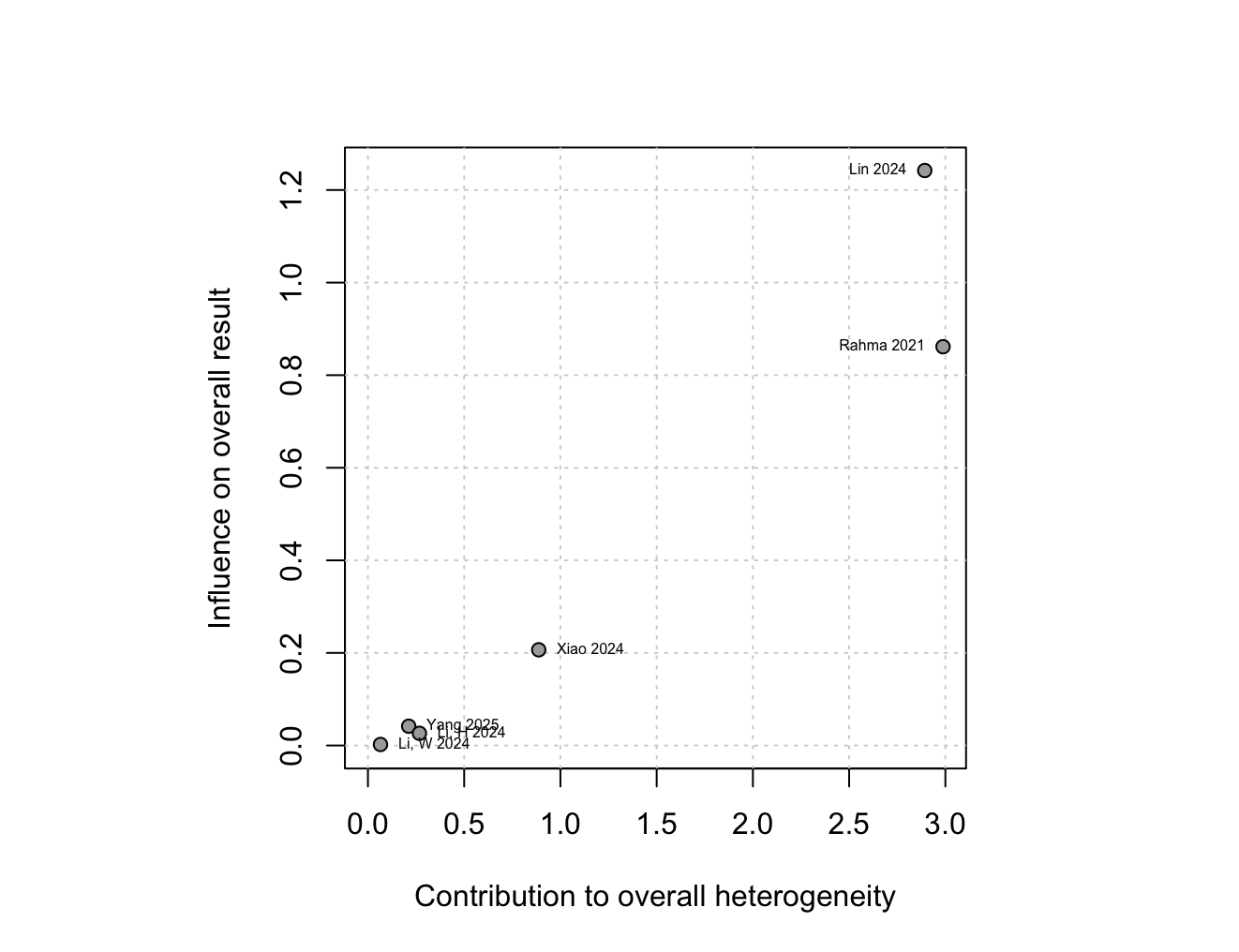


**Supplementary Figure S2:** Baujat plot of pathological complete response (pCR) rate.


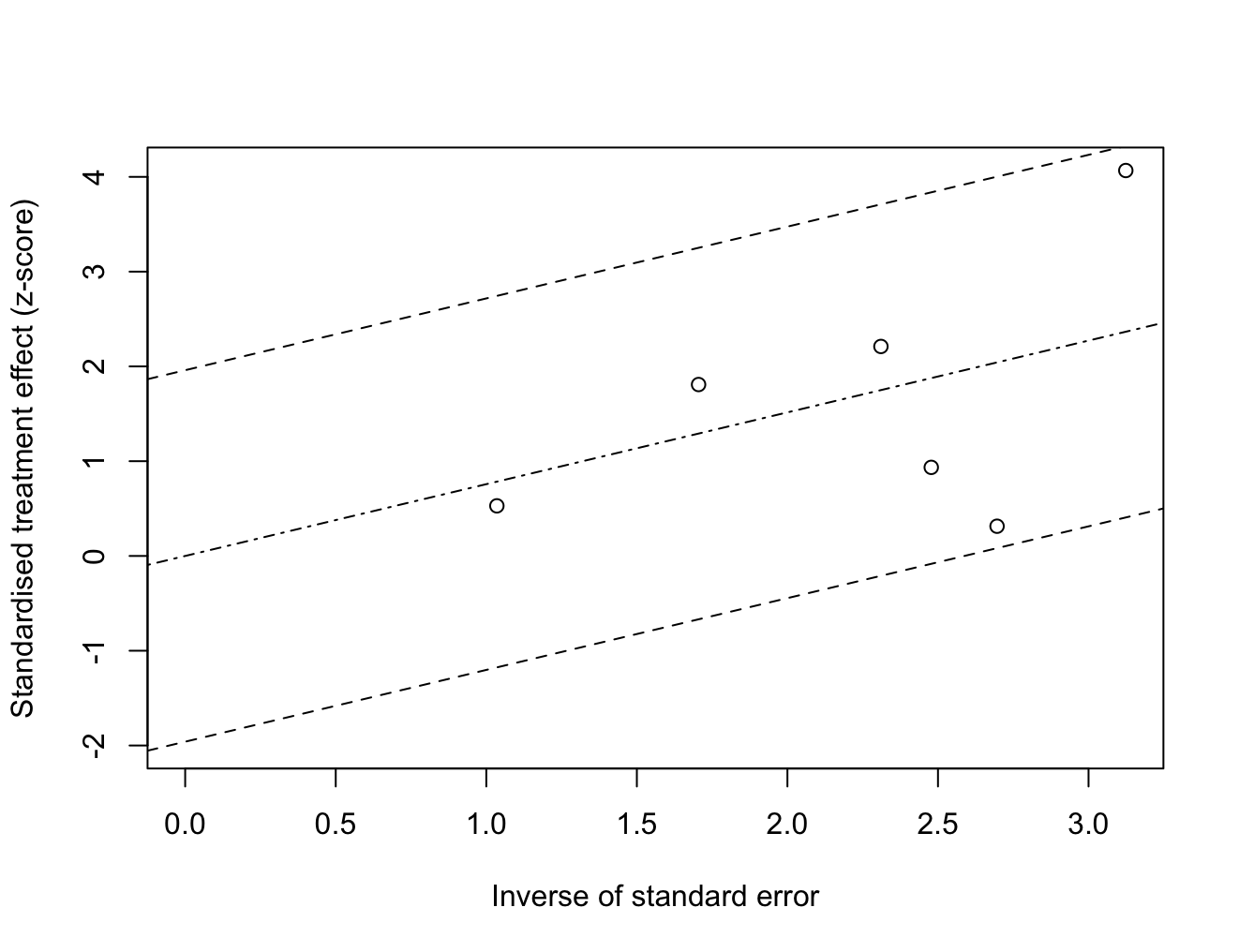


**Supplementary Figure S3:** Galbraith plot of pathological complete response (pCR) rate.


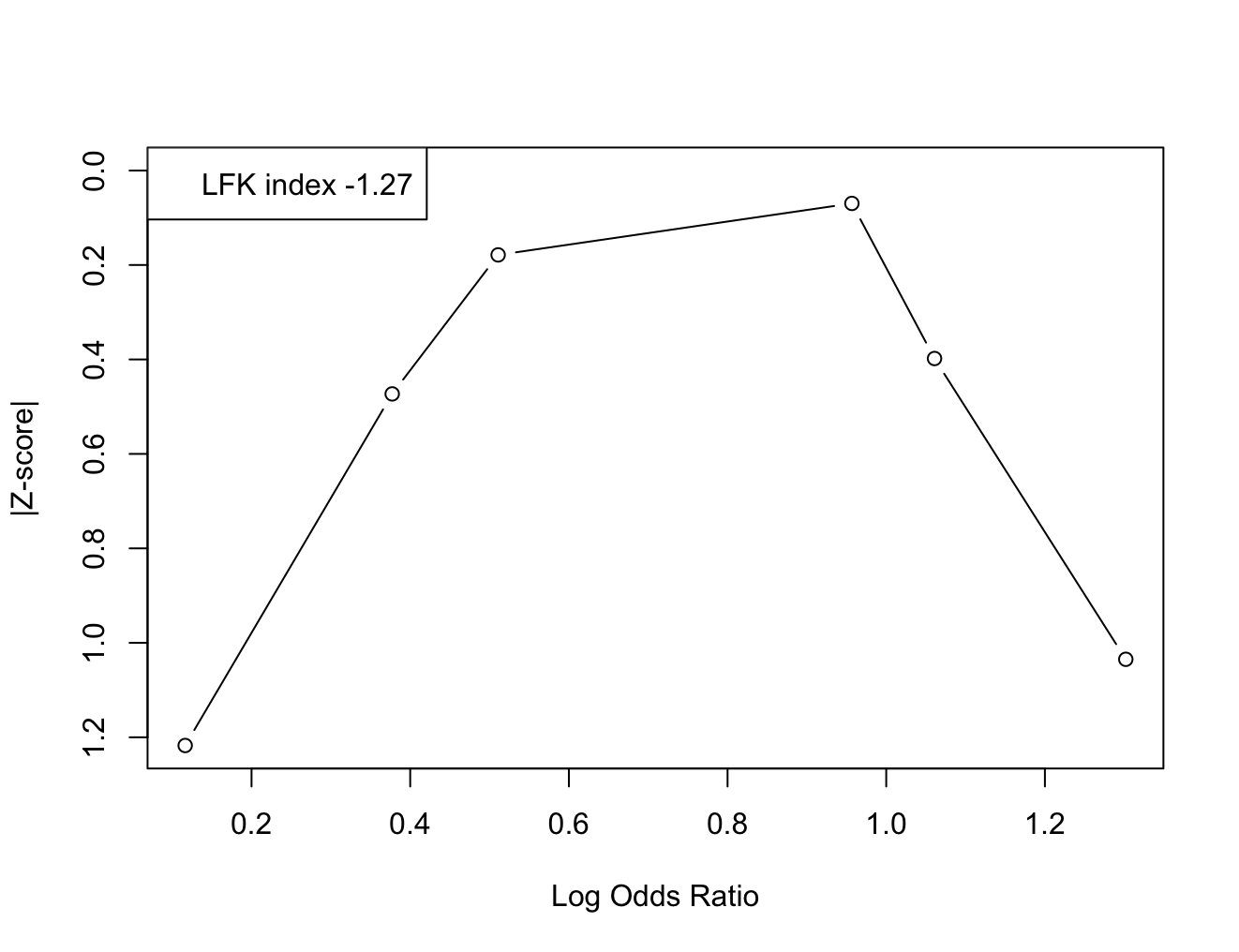


**Supplementary Figure S4:** DOI plot of pathological complete response (pCR) rate.

**
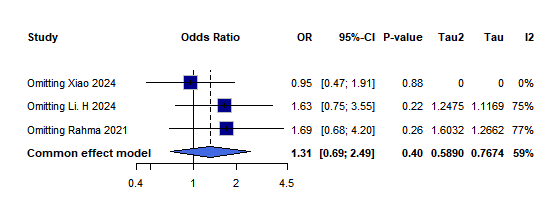
**

**Supplementary Figure S5:** Influence analysis of clinical complete response (cCR) rate.


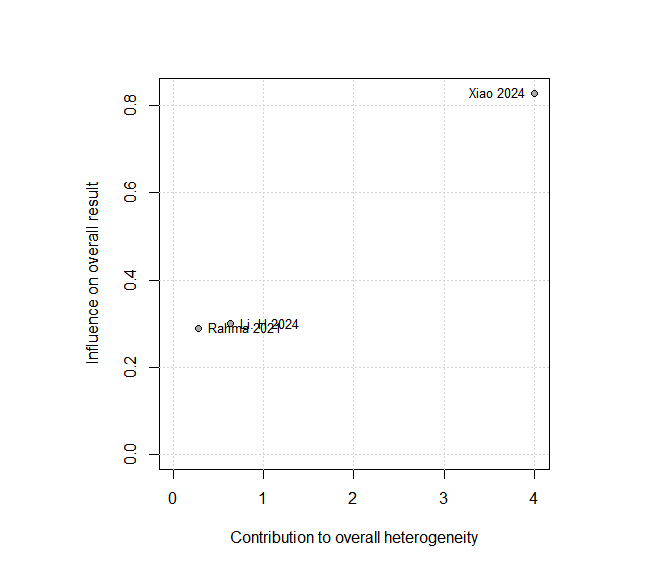


**Supplementary Figure S6:** Baujat plot of clinical complete response (cCR) rate.


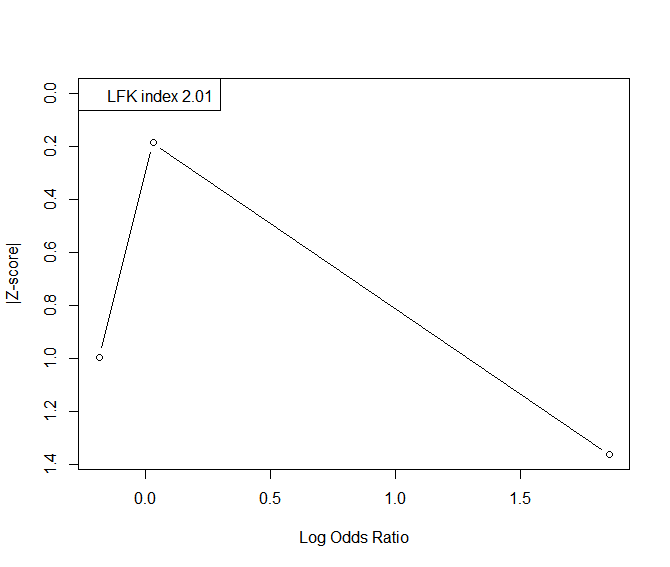


**Supplementary Figure S7:** DOI plot of clinical complete response (cCRR) rate.


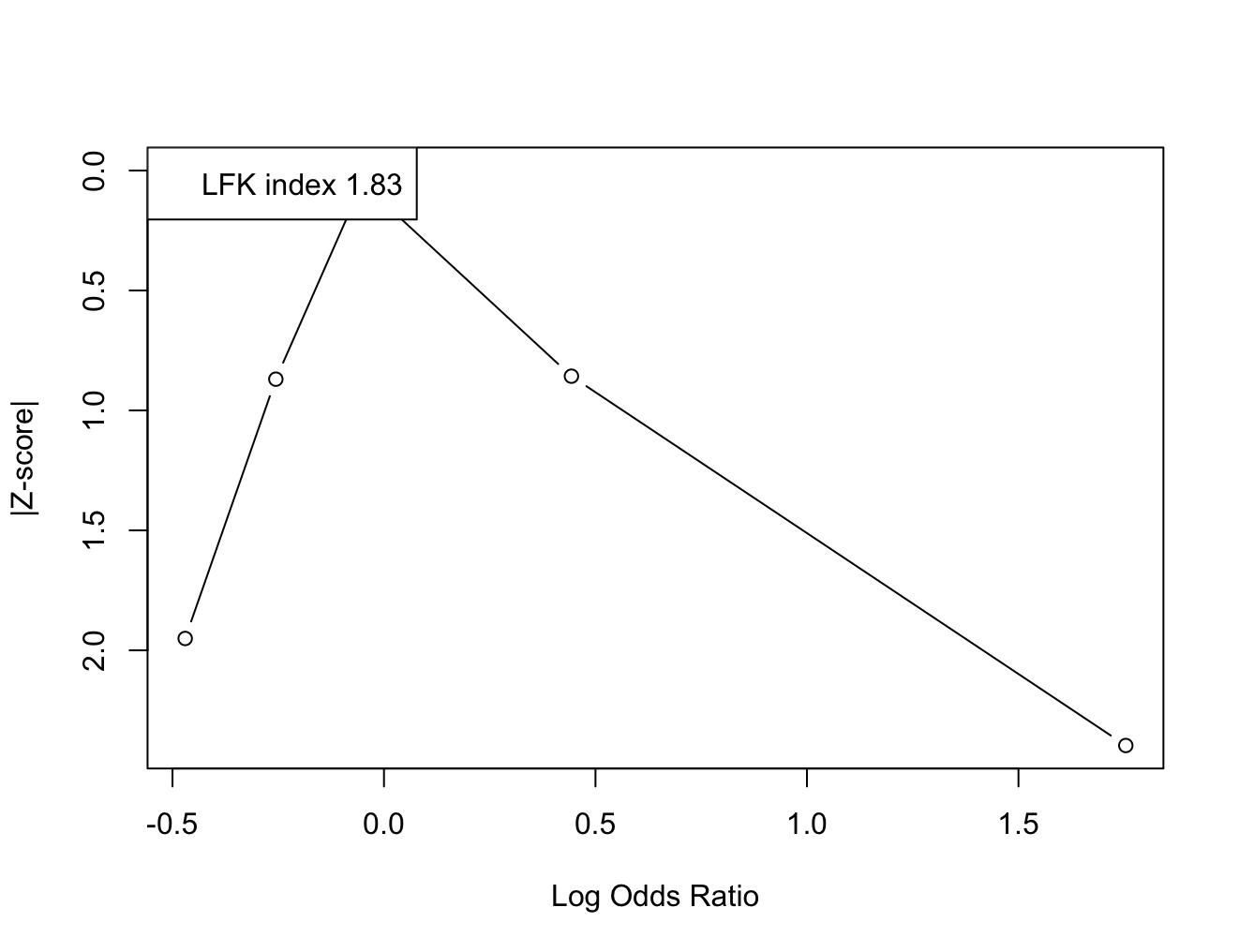


**Supplementary Figure S8:** DOI plot of serious adverse events (SAEs).


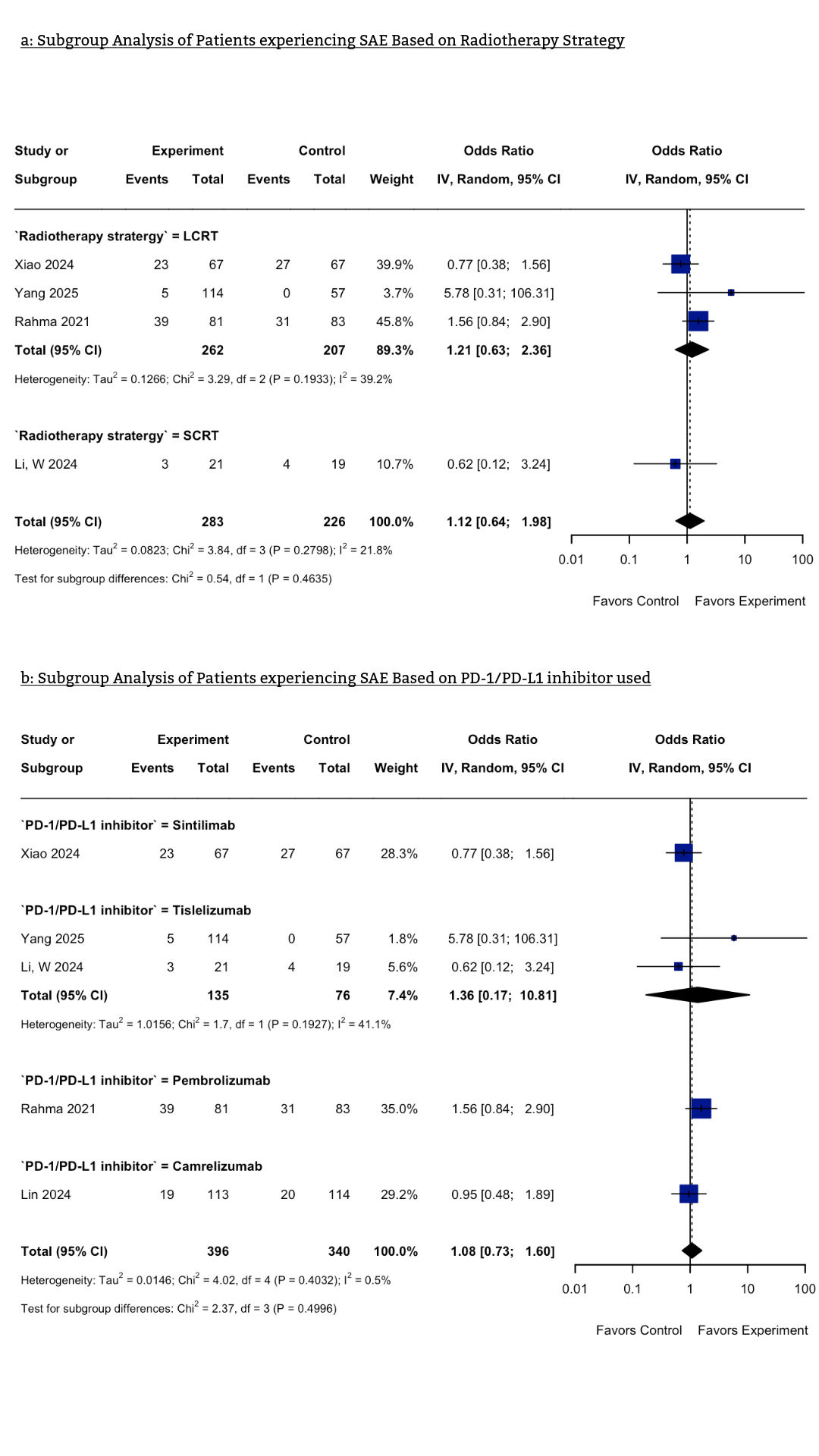


**Supplementary Figure S9:** Subgroup analysis of SAEs based on a: Type of radiotherapy strategy b: PD-1/PD-L1 inhibitor used.

Serious Adverse Event, SAE; CI, Confidence Interval; LCRT, Long-Course Radiotherapy b: SCRT, Short-Course Radiotherapy; PD-1/PD-L1, Programmed cell death protein 1/programmed cell death ligand 1

**Risk of bias of RCTs by Cochrane Rob 2 tool**

| **Study ID** | **D1** | **D2** | **D3** | **D4** | **D5** | **Overall** |  |  |  |
| --- | --- | --- | --- | --- | --- | --- | --- | --- | --- |
| Xiao 2024 |  |  |  |  |  |  |  |  | Low risk |
| Yang 2025 |  |  |  |  |  |  |  |  | Some concerns |
| Li, H 2024 |  |  |  |  |  |  |  |  | High risk |
| Rahma 2021 |  |  |  |  |  |  |  |  |  |
| Li, W 2024 |  |  |  |  |  |  |  |  |  |
| Lin 2024 |  |  |  |  |  |  |  |  |  |
|  |  |  |  |  |  |  |  | D1 | Randomization process |
|  |  |  |  |  |  |  |  | D2 | Deviations from the intended interventions |
|  |  |  |  |  |  |  |  | D3 | Missing outcome data |
|  |  |  |  |  |  |  |  | D4 | Measurement of the outcome |
|  |  |  |  |  |  |  |  | D5 | Selection of the reported result |

**Supplementary Figure S10:** Risk-of-bias summary illustrating review authors' judgments for each risk-of-bias item across all included studies.


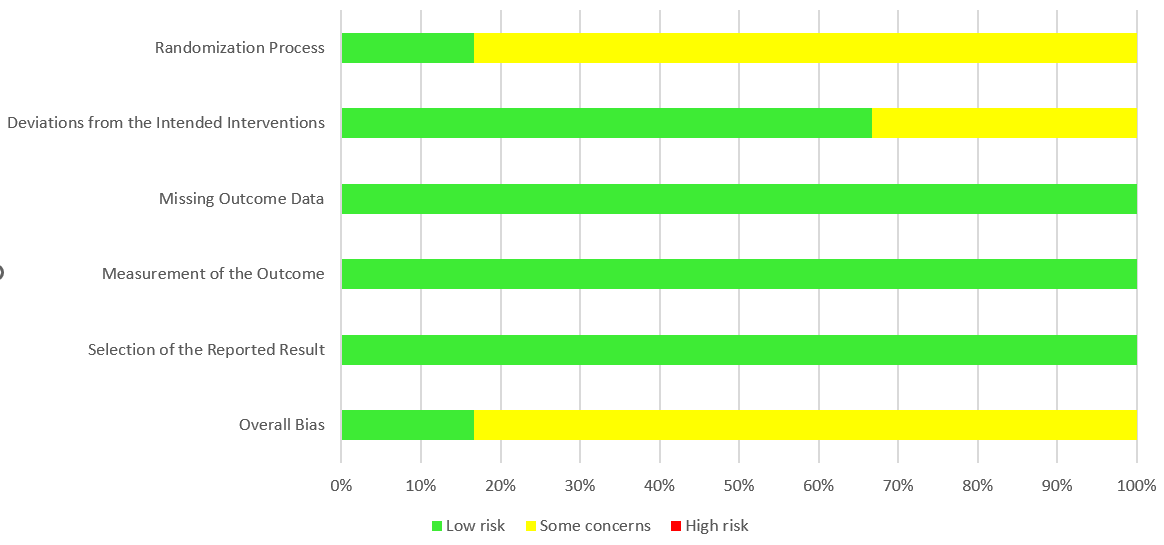


**Supplementary Figure S11:** Risk-of-bias graph illustrating review authors' judgments for each risk-of-bias item, presented as percentages across all included studies.
